# Testing competing factor models of the latent structure of post-traumatic stress disorder and complex post-traumatic stress disorder according to ICD-11

**DOI:** 10.1080/20008198.2018.1457393

**Published:** 2018-04-10

**Authors:** Siobhan Murphy, Ask Elklit, Sarah Dokkedahl, Mark Shevlin

**Affiliations:** a National Centre of Psychotraumatology, University of Southern Denmark, Odense, Denmark; b Psychology Research Institute, Ulster University, Derry, UK

**Keywords:** ICD-11, PTSD, CPTSD, ITQ, CFA, CIE-11, TEPT, TEPT-C, ITQ, CFA, ICD-11, PTSD, CPTSD, ITQ, CFA, • This study examined the factor structure of the International Trauma Questionnaire in a non-Western sample.• A seven-factor first-order correlated model provided the best fit to the data.• Those who met diagnostic criteria for CPTSD reported higher levels of war experiences and psychological problems.

## Abstract

With the publication of the International Statistical Classification of Diseases and Related Health Problems, 11th edition (ICD-11) due for release in 2018, a number of studies have assessed the factorial validity of the proposed post-traumatic stress disorder (PTSD) and complex (CPTSD) diagnostic criteria and whether the disorders are correlated but distinct constructs. As the specific nature of CPTSD symptoms has yet to be firmly established, this study aimed to examine the dimension of affect dysregulation as two separate constructs representing hyper-activation and hypo-activation. Seven alternative models were estimated within a confirmatory factor analytic framework using the International Trauma Questionnaire (ITQ). Data were analysed from a young adult sample from northern Uganda (*n *= 314), of which 51% were female and aged 18–25 years. Forty per cent of the participants were former child soldiers (*n* = 124) while the remainder were civilians (*n* = 190). The prevalence of CPTSD was 20.8% and PTSD was 13.1%. The results indicated that all models that estimated affective dysregulation as distinct but correlated constructs (i.e. hyper-activation and hypo-activation) provided satisfactory model fit, with statistical superiority for a seven-factor first-order correlated model. Furthermore, individuals who met the criteria for CPTSD reported higher levels of war experiences, symptoms of anxiety and depression, and somatic problems than those with PTSD only and no diagnosis. There was also a much larger proportion of former child soldiers that met the criteria for a CPTSD diagnosis. In conclusion, these results partly support the factorial validity of the ICD-11 proposals for PTSD and CPTSD in a non-Western culture exposed to mass violence. These findings highlight that more research is required across different cultural backgrounds before firm conclusions can be made regarding the factor structure of CPTSD using the ITQ.

## Abstract (es)

**Planteamiento**: Con la publicación de la 11ª edición de la Clasificación Internacional de Enfermedades que se publicará en 2018, varios estudios han evaluado la validez factorial de los criterios de diagnóstico propuestos del trastorno por estrés postraumático (TEPT) y trastorno por estrés postraumático complejo (TEPT-C) y si los trastornos están correlacionados pero son constructos distintos.

**Objetivo**: Debido a que la naturaleza específica de los síntomas de TEPT-C aún no se ha establecido con firmeza, este estudio estableció como objetivo examinar la dimensión de la desregulación afectiva como dos constructos separados que representan la hiperactivación y la hipoactivación.

**Métodos**: Se estimaron siete modelos alternativos dentro de un marco analítico factorial confirmatorio usando el Cuestionario Internacional de Trauma (ITQ, por sus siglas en inglés). Los datos se analizaron a partir de una muestra de adultos jóvenes del norte de Uganda (n = 314), de los cuales el 51% eran mujeres y tenían entre 18 y 25 años. El cuarenta por ciento de los participantes eran ex niños soldados (N = 124) mientras que el resto eran civiles (N = 190).

**Resultados**: La prevalencia del TEPT-C fue del 20.8% y la del TEPT fue del 13.1%. Los resultados indicaron que todos los modelos que estimaron la desregulación afectiva como constructos distintos proporcionaron un ajuste satisfactorio del modelo con superioridad estadística para un modelo correlacionado de primer orden de siete factores. Además, las personas que cumplieron con los criterios del TEPT-C indicaron niveles más altos de experiencias de guerra, síntomas de ansiedad y depresión, y problemas somáticos que aquellos con solo TEPT y sin diagnóstico. También hubo una proporción mucho mayor de ex niños soldados que cumplieron con los criterios para un diagnóstico de TEPT-C.

**Conclusiones**: Estos resultados respaldan parcialmente la validez factorial de las propuestas de la CIE-11 para el TEPT y el TEPT-C en una cultura no occidental expuesta a la violencia masiva. Estos hallazgos resaltan que se requiere más investigación en diferentes contextos culturales antes de poder llegar a conclusiones firmes con respecto a la estructura factorial de TEPT-C utilizando el ITQ.

## Abstract (zh)

**背景**: 2018年即将发布的国际疾病分类第11版，许多研究评估了PTSD和CPTSD诊断标准的因子效度，以及这些疾病是否是相关但相异的结果。

**目的**: 由于CPTSD症状的特殊性尚未准确界定，因此本研究旨在考察两种情感调节异常的维度，作为代表过度激活和低激活的两个独立结构。

**方法**: 使用国际创伤问卷（ITQ）在验证性因素分析框架内估计七种替代模型。数据来自乌干达北部（n = 314）的年轻成年人样本，其中51％为女性，年龄在18至25岁之间。 40％的参与者是前童子兵（N = 124），其余的是平民（N = 190）。

**结果**: CPTSD的患病率为20.8％，PTSD的患病率为13.1％。结果表明，估计情感失调作为独特结构的所有模型都表现了令人满意的模型拟合，其中七因子一阶相关模型具有统计优势。此外，符合CPTSD标准的个人报告相较于仅有PTSD和没有诊断结果的个体，表现出较高的战争经历水平、较多的焦虑、抑郁和躯体问题。达到CPTSD诊断标准的前儿童士兵有很大比例。

**结论**: 这些结果在暴露于大规模暴力的非西方文化中部分支持ICD-11关于PTSD和CPTSD提出的因子效度。这些发现强调了，在使用确定ITQ测量的CPTSD的因素结构之前，需要针对不同文化背景进行更多的研究。

## 1. Introduction

The 11th revision of the World Health Organization’s (WHO’s) International Statistical Classification of Diseases and Related Health Problems (ICD-11) is due for release in 2018. This revision is formulated on a public health perspective aiming to improve the clinical utility and the global applicability of diagnoses across a range of socioeconomic and geographic contexts (Forbes et al., 2015). The ICD-11 Working Group for Disorders Specifically Associated with Stress has outlined significant changes to the diagnostic criteria of post-traumatic stress disorder (PTSD), proposing a tripartite model that requires at least one of two re-experiencing symptoms (nightmares or flashbacks experienced in the present), at least one of two avoidance symptoms (internal avoidance or external avoidance associated with the event) and at least one of two hyperarousal symptoms (hypervigilance and exaggerated startle response) representing a sense of current threat.

**Figure 1.**
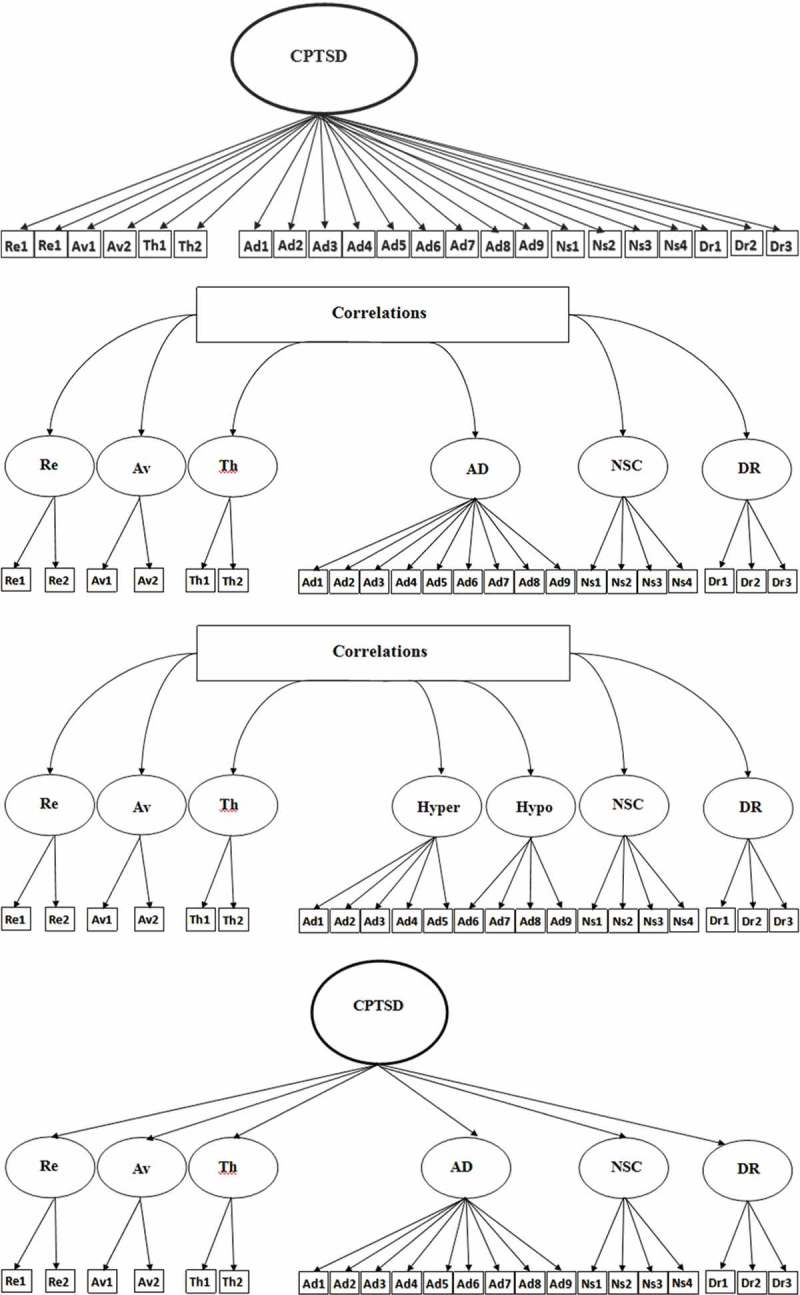
Alternative confirmatory factor analysis (CFA) models. Model 1: unidimensional complex post-traumatic stress disorder (CPTSD). Model 2: six-factor first-order model of CPTSD. Model 3: seven-factor first-order model of CPTSD. Model 4: single-factor second-order with six correlated factors. Model 5: single-factor second-order with seven correlated factors. Model 6: two-factor second-order model with six correlated factors. Model 7: two-factor second-order model with seven correlated factors. Re, re-experiencing; Av, avoidance; Th, sense of threat; Ad, AD, affect dysregulation; Ns, NSC, negative self-concept; Dr, DR, disturbed relationships; Hyper, hyper-activation; Hypo, hypo-activation; PTSD, post-traumatic stress disorder.

**Figure 1.**
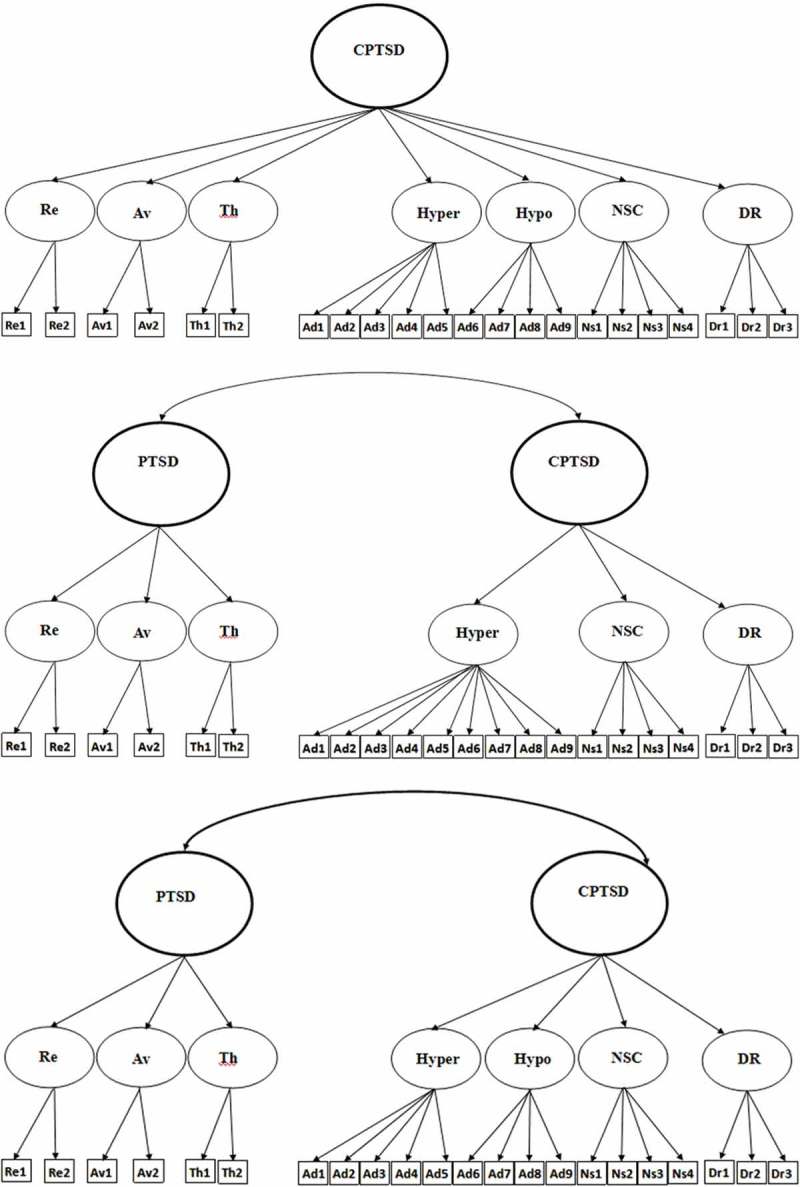
(Continued).

These symptoms define PTSD as a response characterized by some degree of fear or horror related to a specific traumatic event. The proposed revisions also are intended to improve diagnostic accuracy by directing clinicians to the core PTSD symptoms, thereby reducing comorbidity with other traumatic responses, and use functional impairment rather than a specific traumatic experience to determine the diagnostic threshold (Cloitre, Garvert, Brewin, Bryant, & Maercker, 2013; Maercker et al., 2013). The proposed reduction in the diagnostic criteria of PTSD contrasts significantly with the 20 symptoms across four domains – intrusions, avoidance, negative alternations in cognitions and mood, and alternations in arousal and reactivity – in the Diagnostic and Statistical Manual of Mental Disorders, fifth edition (DSM-5) (American Psychiatric Association, 2013). Several studies have examined the implications of these different conceptualizations of PTSD by examining prevalence estimates derived from the ICD-11 and DSM-5 diagnostic classifications, and found that ICD-11 produces significantly lower prevalence rates than DSM-5 (Hansen, Hyland, Armour, Shevlin, & Elklit, 2015; O’Donnell et al., 2014; Wisco et al., 2016).

Another proposed revision to the ICD-11 is the addition of a ‘sibling’ disorder, complex post-traumatic stress disorder (CPTSD) (Cloitre et al., 2013; Maercker et al., 2013). CPTSD symptoms manifest following prolonged and repeated traumatic events (e.g. war captivity, genocide and childhood sexual abuse) and reflect disturbances in self-organization (DSO). CPTSD consists of the three core features of PTSD in addition to difficulties in affect dysregulation (AD), self-concept and relational functioning. A diagnosis of CPTSD requires that in addition to meeting PTSD diagnostic criteria, at least one symptom from each of the DSO domains must be present (Maercker et al., 2013). AD encompasses a range of symptoms resulting from difficulties in emotion regulation, which may manifest as heightened emotional reactivity (hyper-activation) or as a lack of emotions or dissociative symptoms (hypo-activation). Self-concept difficulties refer to persistent negative self-beliefs, feelings of worthlessness and guilt. Disturbances in relational functioning are characterized by difficulties in feeling emotionally close to or engaging with others. A distinguishable feature of the two trauma-related disorders is that PTSD symptoms are related to the trauma-specific stimuli, whereas DSO symptoms are ubiquitous and occur across various contexts and relationships regardless of proximity to traumatic reminders (Cloitre et al., 2013). Given that a PTSD diagnosis is a requirement for the diagnostic criteria of CPTSD, it imposes a hierarchical relationship between the two constructs.

Emerging evidence supports the factorial validity of both ICD-11 PTSD (e.g. Hansen et al., 2015; Hyland, Brewin, & Maercker, 2017; O’Donnell et al., 2014) and CPTSD (Hyland et al., 2017; Shevlin et al., 2017; Tay, Rees, Chen, Kareth, & Silove, 2015). However, given that this is a nascent area of research, these studies are limited by the use of proxy items derived from different scales that measure PTSD and proposed DSO symptoms. Furthermore, universal agreement on the defined set of symptoms has yet to be established and no published standardized measurement tool exists, which makes it difficult to evaluate the nature of the construct (Bryant, 2012). Therefore, there is a clear research need for a consistent definition of CPTSD so that the proposed construct can be understood clearly in relation to other post-traumatic reactions and to examine the key mechanisms that may underlie the disorder. To overcome this limitation, the International Trauma Questionnaire (ITQ) was developed in line with the proposed diagnostic criteria of PTSD and CPTSD (Cloitre, Roberts, Bisson, & Brewin, 2015). Although this measure is still in the development stages in terms of refining the symptom content and factor structure, a few studies using clinical and non-clinical samples have assessed the distinctiveness of PTSD and CPTSD using this instrument (Ben-Ezra et al., 2018; Hyland et al., 2017; Karatzias et al., 2016).

The factorial validity of PTSD and CPTSD has been tested, with emerging evidence supporting three possible models: a factor structure in which all six symptom clusters are correlated with each other in a non-hierarchical fashion (Tay et al., 2015), a single higher-order factor supported by six second-order factors (Silove, Tay, Kareth, & Rees, 2017), and a second-order two-factor model represented by PTSD and DSO and six correlated first-order factors (Hyland et al., 2017; Karatzias et al., 2016; Shevlin et al., 2017). Collectively, the evidence indicates the superiority of the two-factor higher-order model comprised of the PTSD and DSO domains, which supports the theoretical model of CPTSD as being comprised of two distinct but related components.

The factor structure of the ITQ has been tested using a nationally representative sample of Israeli adults (Ben-Ezra et al., 2018). This study, however, tested the dimensionality of the affective dysregulation domain as distinct factors representing hyper-activation and hypo-activation. The results indicated that a both a correlated first-order model with three PTSD and four DSO domains (affect dysregulation distinguishing between hyper- and hypo-activation symptoms) and a two-factor second-order model, PTSD and DSO, both fitted the data well, with the correlated seven-factor model providing superior fit. These findings indicated that hyper-activation and hypo-activation indicators were better represented by two correlated latent variables (*r* = 0.72) rather than one latent variable. This highlights that more research attention on the dimensionality of AD is warranted. Furthermore, while emotional dysregulation has been previously considered a unitary construct, evidence is emerging that different types of emotional dysregulation are associated with different types of childhood abuse (Weiss, Tull, Lavender, & Gratz, 2013).

The current study aimed first to employ confirmatory factor analysis (CFA) to test the factorial validity of PTSD and CPTSD using the ITQ, and also to test the AD domains of hyper- and hypo-activation symptoms as separate factors using a sample of young adults from northern Uganda. This sample is ideally placed to evaluate the factor structure of CPTSD as they were all children during the decade of civil conflict in northern Uganda (1995–2006) and were exposed and subjected to severe civil rights violations and extreme violence. The second aim was to assess whether PTSD and CPTSD diagnoses are differentially associated with demographic and trauma variables. The current study is predicated on the following hypotheses. First, it was hypothesized that factorial models of the ITQ which discriminate between PTSD and DSO symptoms would produce good model fit (Hyland et al., 2017; Karatzias et al., 2016; Shevlin et al., 2017). Secondly, based on findings from Ben-Ezra et al. (2018), it was hypothesized that modelling the AD dimension of hyper-activation and hypo-activation as correlated but distinct constructs would provide a better fit than models estimating AD as a unitary construct. Thirdly, consistent with previous evidence demonstrating that CPTSD is associated with greater psychopathology (Elklit, Hyland, & Shevlin, 2014; Perkonigg et al., 2016), it was hypothesized that CPTSD would be associated with higher levels of psychological problems than those with PTSD only and no diagnosis.

## 2. Methods

### 2.1. Sample

This study was conducted in Gulu, in the subcounty of Awach, the biggest district of northern Uganda. The sample (*N* = 314) included both males (49%) and females (51%) aged between 18 and 25 years with a mean age of 22.30 (SD = 2.84) years. Almost 40% (39.5%) of the participants had been abducted by the Lord’s Resistance Army (LRA) as child soldiers (*n* = 124), while the remaining participants were civilians (*n* = 190).

The lack of a national register in Uganda made random sampling difficult. The local leaders of the four parishes in Awach were informed of the study and selected the participants based on the principles of random sampling and in accordance with the size of the parish. Participants were further selected equally from each village within the parish, with equal participation according to gender, age, former child soldiers and civilians. This allowed for a broad distribution of participants within the Awach community. Exclusion criteria were the presence of psychotic symptoms or individuals who were unable to complete the interview owing to mental disability.

Participants provided written consent for their participation and those who could not write were asked to sign with their thumbprint in ink. Before giving consent, the participants were informed about the content of the study, their rights to decline and withdraw at any time, and the confidentiality of their participation in the study. None of the participants who were selected declined to participate. As per agreement with Victim’s Voice (VIVO), participants who fulfilled the criteria for PTSD, using the ICD-11 measure, and who wished to receive help were referred for counselling at VIVO. Participants were informed that the doctor at the local health centre had agreed that all participants could seek help and support at the health centre if necessary. The Institutional Review and Ethics Committee at the Lacor Hospital in Gulu approved the project along with the translations. All measures and instruments were translated and back-translated from English into Luo, the local language of the Awach community.

The questionnaires were read out loud for the participants to avoid any possible reading disabilities in the rural areas of northern Uganda. Local field assistants asked the questions in Luo. The interviews took place at the homes of the participants.

### 2.2. Measures

#### 2.2.1. UNICEF War Trauma Screening Scale (UNICEF, 2010)

Trauma exposure was measured using the United Nations Children’s Fund (UNICEF) War Trauma Screening Scale, which was originally developed for Bosnia and Herzegovina but has been adapted for use in African war-affected youth (Amone-P’Olak et al., 2013). The instrument consists of items relating to personal injury (six items), witnessing violence (11 items), injuries and threats to self (five items), deaths (seven items), physical threats to loved ones (four items), material losses (four items), harm to loved ones (four items), separation (two items), displacement (five items), participating in armed groups (four items) and sexual abuse (three items). In the current study, the reliability estimate for the total scale was high (*α* = 0.93).

#### 2.2.2. International Trauma Questionnaire (ITQ) Cloitre et al., (2015)

The ITQ is a 23-item self-report measure for ICD-11 PTSD and CPTSD diagnoses that is currently under development. The measure corresponds to the three clusters of PTSD: re-experiencing (RE) (items P1 and P2); avoidance (AV) (items P3 and P4); and sense of threat (Th), which is manifested as increased arousal and hypervigilance (items P5 and P6). CPTSD is measured through the inclusion of 16 symptoms that capture DSO symptoms. These items form four clusters, with two relating to affect regulation characterized by hyper-activation (AD1–AD5) or hypo-activation (AD6–AD9), one cluster relating to negative self-concept (NSC10–NSC13), and one to disturbed relationships (DR14–DR16).

The response format corresponds to the degree to which the symptoms bothered the individual in the past month and are scored on a Likert scale ranging from 0 (not at all) to 4 (extremely). The scale can be used to generate a self-report ICD-11 PTSD or CPTSD diagnosis. A diagnosis of PTSD requires a score of ≥2 for at least one symptom in each of its three clusters. A diagnosis of CPTSD requires meeting the criteria for PTSD and the following scores for each of the three DSO clusters. AD requires a score ≥10 on items 1–5 (hyper-activation) or a score of ≥8 on items 6–9 (hypo-activation); for the NSC items a score ≥8 and for DR a score ≥6 are required. In the current study, the reliability estimates were adequate, with Cronbach’s alpha for the total scale (*α* = 0.91), hyper-activation (*α* = 0.73), deactivation (*α* = 0.75), negative self-concept (*α* = 0.83) and relational disturbance (*α* = 0.79).

#### 2.2.3. African Youth Psychosocial Assessment (APAI) (Betancourt et al., 2009)

Mental health was assessed by the APAI, which is a field-based instrument developed for use in northern Uganda. This measure comprises four subscales of depression/anxiety (18 items), somatic complaints (three items), conduct problems (10 items) and prosocial behaviour (five items). The instrument is measured on a four-point Likert scale ranging from 0 (not at all) to 3 (always). The reliability in the current sample was adequate, with Cronbach’s alpha values for anxiety/depression (*α* = 0.91), conduct problems (*α* = 0.81), somatic complaints (*α* = 0.66) and prosocial behaviour (*α* = 0.64).

### 2.3. Statistical analysis

Seven alternative factor models of the ITQ were estimated using CFA. Model 1 is a unidimensional model where all symptoms load on the single latent variable CPTSD. Model 2 is a correlated first-order six-factor model (Re, Av, Th, AD, NSC and DR). Model 3 is a correlated first-order seven factor model that treats AD symptoms measuring hyper-activation (hyper; AD1–AD5) and hypo-activation (hypo; AD6–AD9) as distinct constructs. Model 4 is a single second-order model (CPTSD) with six first-order factors that tests whether the covariation of the six first-order factors can be explained by a single CPTSD factor. Model 5 a single second-order model (CPTSD) with seven first-order factors. Model 6 specified two correlated second-order factors (PTSD and DSO) to explain the covariation among six first-order factors. Model 7 was similar to Model 6 but separated the AD symptoms into the hyper- and hypo-activation dimensions. These models are illustrated in Figure 1.

To assess the goodness of fit for each model a range of fit statistics was examined, including the comparative fit index (CFI) (Bentler, 1990), the Tucker–Lewis Index (TLI) (Tucker & Lewis, 1973), a non-significant chi-squared statistic (*χ*^2^) and the root mean square error of approximation (RMSEA) (Steiger, 1990). Specifically, a CFI/TLI above 0.90 is indicative of acceptable model fit. In addition, an RMSEA value below 0.08 indicates a reasonable error of approximation.

Subsequent analyses were conducted to examine the association between diagnostic status and total war experiences, and the four subscales of the APAI (anxiety–depression, conduct problems, somatic problems and prosocial behaviour). A categorical variable representing diagnostic status was created to examine differences between CPTSD, PTSD only and no diagnosis on these variables.

## 3. Results

### 3.1. Sample characteristics

A combined prevalence of 33.9% (*n* = 106) of the sample met the ICD-11 criteria for PTSD or CPTSD using the ITQ, with *n *= 65 (20.8%) meeting the criteria for CPTSD, *n *= 41 (13.1%) for PTSD only and *n *= 206 (66.1%) receiving no diagnosis. Bivariate associations indicated no significant difference between gender and diagnostic status (*χ*^2^(2) = 2.28, *p* > 0.05). Significant associations were observed for diagnostic status and being a former child soldier (*χ*^2^(2) = 16.32, *p* < 0.001), with 61% of former child soldiers meeting the threshold for CPTSD, 39% PTSD and 32.7% no diagnosis. Table 1 shows the endorsement rates for the individual PTSD and DSO symptom clusters. The results indicate that 73.5% met the criteria for the re-experiencing factor, and hyper-activation was the most commonly endorsed DSO factor (79.6%). Females reported higher levels of re-experiencing, sense of current threat, hypo-activation and NSC symptoms than males; however, there were no significant gender differences in any symptom cluster.

**Table 1.** Descriptive statistics of International Trauma Questionnaire (ITQ) subscales and gender.

ICD-11	Male (%)	Female (%)	Total (%)	*χ*^2^ (df) *p*
Re-experiencing	46.7	53.3	73.5	1.84 (1) 0.175
Avoidance	50.7	49.3	66.3	0.85 (1) 0.357
Sense of threat	48.9	51.1	59.7	0.00 (1) 0.961
Hyper-activation	51.2	48.8	79.6	2.28 (1) 0.131
Hypo-activation	48.4	51.6	49.5	0.05 (1) 0.819
Negative self-concept	47.9	52.1	54.0	0.18 (1) 0.668
Disturbed relationships	50.5	49.5	33.3	0.16 (1) 0.689

### 3.2. CFA results

The fit statistics for the seven models of PTSD and CPTSD are presented in Table 2. Models 3, 5 and 7 all demonstrated adequate fit to the data. Notably, the results indicate that all models that treated the AD dimension as distinct hyper-activation and hypo-activation domains provided superior fit to the data. All chi-squared statistics were statistically significant; however, this should not lead to the rejection of the models as the power of the chi-squared test is positively related to sample size (Tanaka, 1987). The model fit indices for these three models were similar, therefore chi-squared difference tests were conducted to examine model superiority. These tests indicated that Model 3 was significantly better fitted to the data than Model 5 (*χ*^2^(14) = 72.93, *p* < 0.001) and Model 7 (*χ*^2^(13) = 61.47, *p* < 0.001). Model 7 was significantly better than Model 5 (*χ*^2^(1) = 1.02, *p* < 0.001). Model 3 therefore was considered the best fitting model. The standardized factor loadings and factor correlations for this model are reported in Table 3.

**Table 2.** Fit statistics for the alternative models of the ICD-11 post-traumatic stress disorder (PTSD) and complex post-traumatic stress disorder (CPTSD) symptoms.

Model	Model	*χ*^2^	df	CFI	TLI	RMSEA	WRMR
1	Unidimensional	1426.36	209	0.845	0.829	0.136	2.05
						(0.130**–**0.143)	
2	Correlated six-factor first-order	1050.10	194	0.891	0.870	0.119	1.65
						(0.112**–**0.126)	
**3**	Correlated seven-factor	**737.92**	**188**	**0.930**	**0.914**	**0**.**097**	**1.35**
	first-order					**(0.089–0**.**104)**	
4	One-factor second-order, six first-order factors	1060.01	203	0.891	0.876	0.097	1.72
						(0.109**–**0.123)	
**5**	One-factor second-order, seven first-order factors	**778.33**	**202**	**0.927**	**0.916**	**0**.**095**	**1.46**
						**(0.088–0.103)**	
6	order, six first-order factors	1053.59	202	0.892	0.876	0.116	1.70
						(0.109**–**0.123)	
**7**	Two-factor second-order, seven first-order factors	**767.94**	**201**	**0.928**	**0.917**	**0.095**	**1.44**
						**(0.088–0**.**102)**	

df, degrees of freedom; CFI, comparative fit index; TLI, Tucker–Lewis Index; RMSEA, root mean square error of approximation; WRMR, weighted root mean square residual.

**Table 3.** Standardized factor loadings and factor correlations for the seven-factor first-order complex post-traumatic stress disorder (CPTSD) model.

	Re	Av	Th	Hyper	Hypo	NSC	DR
Re1	0.71						
Re2	0.85						
Av1		0.66					
Av2		0.76					
Th1			0.88				
Th2			0.90				
AD1				0.60			
AD2				0.80			
AD3				0.79			
AD4				0.79			
AD5				0.50			
AD6					0.51		
AD7					0.58		
AD8					0.80		
AD9					0.82		
NSC1						0.89	
NSC2						0.88	
NSC3						0.71	
NSC4						0.76	
DR1							0.81
DR2							0.83
DR3							0.73
Factor correlations							
Av	0.66	–					
Th	0.76	0.35	–				
Hyper	0.63	0.41	0.42	–			
Hypo	0.81	0.64	0.76	0.66	–		
NSC	0.69	0.35	0.67	0.64	0.85	–	
DR	0.71	0.42	0.71	0.54	0.91	0.83	–

Re, re-experiencing; Av, avoidance; Th, sense of threat; AD, affect dysregulation; NSC, negative self-concept; DR, disturbed relationships; Hyper, hyper-activation; Hypo, hypo-activation.

The association between diagnostic status and mean total trauma experiences was significant [*F*(2, 309) = 15.60, *p* < 0.001] for CPTSD (39.58, SD = 7.38), PTSD (36.49, SD = 8.37) and no diagnosis (32.67, SD = 9.56). The mean APAI anxiety–depression subscale also differed significantly [*F*(2,310) = 55.16, *p* < 0.001] across CPTSD (32.02, SD = 9.25), PTSD (25.68, SD = 7.63) and no diagnosis group (18.81, SD = 9.30). The mean APAI somatic subscale also differed significantly [*F*(2,309) = 21.45, *p* < 0.001] across CPTSD (5.91, SD = 2.20), PTSD (4.80, SD = 2.27) and no diagnosis (3.91, SD = 2.15). The prosocial and conduct problems subscales did not differ significantly across diagnostic status.

## 4. Discussion

The primary aim of this study was to test alternative models of the factor structure of the ITQ for CPTSD. The results from the CFA indicated that a correlated first-order model (Model 3) with the three PTSD domains and the four DSO domains (hyper, hyper, NSC and DR) was the best fitting model. The model that included two second-order latent variables, PTSD and DSO (Model 7), to explain the covariation among the seven first-order factors also fitted the data; however, the chi-squared difference test indicated that Model 3 provided a significantly better fit to the data. Previous studies have found mixed support for the alternative factor models, with some evidence indicating model superiority for a second-order two-factor model with six correlated first-order factors (Hyland et al., 2017; Karatzias et al., 2016; Nickerson et al., 2016) and others finding support for a higher-order one-factor model representing CPTSD and six second-order factors (Silove et al., 2017). However, an important difference between these studies and the current study is that the AD factor was estimated as a unidimensional construct and the study conducted by Silove and colleagues did not estimate a correlated first-order model. The current results supporting that affect dysregulation is better represented as two correlated but distinct factors (*r* = 0.66) are consistent with a study conducted on a community sample of Israelis, which found that a correlated first-order seven-factor model with hyper- and hypo-activation modelled as correlated but distinct constructs was the best fitting model (Ben-Ezra et al., 2018).

Furthermore, the finding that treating AD as distinct factors provided superior fit to all models that treated them as a unitary construct highlights an important area for future research. It has been well established that problems with emotional dysregulation are a common consequence of trauma across a variety of traumatized populations (Dvir, Ford, Hill, & Frazier, 2014; Messman-Moore & Bhuptani, 2017). However, evidence suggests that different aspects of affect dysregulation may be more salient in certain types of trauma. For example, symptoms that fall into the hyper-activation domain include uncontrollable anger, which has been found to have a relatively low endorsement in adult survivors of child sexual abuse (Cloitre, Garvert, Weiss, Carlson, & Bryant, 2014), whereas it has been found to be highly endorsed in survivors of mass conflict and severe human rights violations (Hinton, Hsia, Um, & Otto, 2003; Murphy, Elklit, Dokkedahl, & Shevlin, 2016; Rees et al., 2013). Moreover, in a study of survivors of an industrial disaster, anger was the only symptom that increased over a period of 30 months (Weisæth, 1984).

The prevalence of CPTSD in the current sample remains high (20.8%) despite the cessation of hostilities being in place for over 10 years. This finding is considerably higher than reported by Silove et al. (2017) and Ben-Ezra et al. (2018), with CPTSD prevalence estimates of 3% and 2.6%, respectively, but lower than a multicultural refugee sample of whom 32.8% met the CPTSD criteria (Nickerson et al., 2016). However, given the context of the traumatic exposure that these individuals faced during their childhood, this finding is not surprising. Furthermore, the results indicated that 61% of those who were abducted by the LRA met the threshold for CPTSD compared to 39% who met the threshold for PTSD. This finding was not unexpected as child soldiers represent a population likely to experience CPTSD due to exposure to a wide range of traumatic experiences such as torture, killings and sexual abuse at an early age. Child soldiers are also often abducted into armed groups such as the LRA against their wishes and attempts to escape are accompanied by severe physical attacks or death. Former child soldiers have the additional burden of being both recipients and perpetrators of violence (Amone-P’Olak, Dokkedahl, & Elklit, 2017), with studies showing greater difficulties in psychological recovery and reintegration into the community (Amone-P’Olak, Elklit & Dokkedahl, 2017; Bayer, Klasen, & Adam, 2007), which ultimately may impede trauma recovery.

The results indicated that gender was not significantly associated with meeting the criteria for CPTSD, PTSD or any of the individual symptom clusters. This finding is consistent with previous studies (Cloitre et al., 2013; Hyland et al., 2017; Karatzias et al., 2016; Wolf et al., 2015). A possible explanation may be that the current sample was exposed to very high levels of war experiences and massive human rights violations, which may account for the non-significant effect of gender. The findings also indicated that those with CPTSD had significantly higher means for total war experiences, anxiety–depression and somatic problems compared to the PTSD-only and no diagnosis groups. This is consistent with previous studies which demonstrated that CPTSD is associated with higher levels of psychopathology and comorbid conditions (Elklit et al., 2014; Perkonigg et al., 2016).

The results of this study should be considered in the light of some limitations. First, the ITQ is still under development regarding the precise symptoms, particularly for symptoms measuring DSO. Therefore, more research is needed to assess the psychometric properties of this scale, particularly regarding the AD dimension. Secondly, the current findings did not include indicators of functional impairment, which is a requirement for ICD-11 PTSD and CPTSD; therefore, the estimates derived in this study should be considered with this in mind. Thirdly, the current findings are based on a sample of young adults from northern Uganda, and it is unknown how they will generalize to other populations. Finally, a criticism with the CPTSD construct is that some argue that it is PTSD with comorbid borderline personality disorder (BPD) due to symptom overlap, particularly in regard to emotional dysregulation, which we did not address in the current study. However, findings from a latent class analysis revealed qualitatively distinct CPTSD and BPD profiles that were distinguishable on key BPD indicators, such as fear of abandonment and self-harming behaviours (Cloitre et al., 2014). Furthermore, while both conditions include DSO symptoms, BPD, for example, is typically characterized by an unstable sense of self that can alternate between highly positive and negative self-evaluation, whereas CPTSD is characterized by a stable negative sense of self (Brewin et al., 2017). Finally, the sample size in the current study was relatively low, and therefore replication in larger samples is warranted.

In conclusion, the findings reported in the current study indicated that PTSD and DSO symptom clusters were multidimensional rather than hierarchical among a young adult sample of traumatized civilians and former child soldiers from northern Uganda. This study also provides support for the separation of the affective dysregulation dimension into distinct hyper- and hypo-activation domains. These findings add to a growing body of evidence regarding the ICD-11 proposals for PTSD and CPTSD; however, it is evident that more research is needed before determining the nature and number of DSO symptoms that are required for a diagnosis. Importantly, this study has shown the international applicability of both PTSD and CPTSD in a non-Western sample, which is one of the goals of the ICD-11. The current results also illustrate that a decade since the conflict in northern Uganda ended there remains a high proportion of young adults who are exhibiting symptoms associated with CPTSD. These findings have important treatment implications, as without acknowledging the distinctiveness between more complex forms of traumatic response, individuals may not receive the appropriate treatment. For example, a randomized controlled trial on a sample of childhood abuse survivors found evidence that staged treatment, addressing DSO symptoms of affect dysregulation and problems in interpersonal relationships, prior to engaging in trauma-focused exposure, was more effective than an exposure treatment without skills training and a non-exposure skills condition (Cloitre et al., 2010). The results of this study underscore the importance of recognizing CPTSD in treatment settings and highlight that more research is needed to assess the validity of the ITQ across different traumatized populations and cultural settings.
